# Supplementary material for: Pollution Assessment Based on Element Concentration of Tree Leaves and Topsoil in Ayutthaya Province, Thailand
Source: Int J Environ Res Public Health. 2020 Jul 17;17(14):5165. doi: 10.3390/ijerph17145165 (PMC7400151; doi:10.3390/ijerph17145165)
Supplement: Supplementary file 1 [file ijerph-17-05165-s001.pdf]

International Journal of
Environmental Research and Public Health

MDPI

Supplementary Table 1. Result of Generalized Linear Model (GLZ) of elemental concentration of tree leaves.

Elements		Areas (Industry, Urban and Rural)	Tree Species
Al	LogLikelihood	−219.558	−205.565
	p	0.006	<0.001
	Chi^2	10.295	27.988
Ba	LogLikelihood	−113.926	−103.181
	p	<0.001	<0.001
	Chi^2	18.136	21.49
Ca	LogLikelihood	−393.405	−375.591
	p	0.221	<0.001
	Chi^2	3.023	35.629
Cr	LogLikelihood	−41.005	−29.523
	p	0.022	<0.001
	Chi^2	7.665	22.963
Cu	LogLikelihood	−98.713	−66.149
	p	0.276	<0.001
	Chi^2	2.577	65.127
Fe	LogLikelihood	−215.215	−204.805
	p	0.003	<0.001
	Chi^2	11.896	20.82
Mg	LogLikelihood	−311.848	−302.595
	p	0.274	<0.001
	Chi^2	2.586	18.506
Mn	LogLikelihood	−204.238	−198.77
	p	0.564	0.004
	Chi^2	1.144	10.936
Na	LogLikelihood	−269.918	−264.514
	p	0.092	0.004
	Chi^2	4.764	10.808
Ni	LogLikelihood	−14.878	−9.054
	p	0.441	0.003
	Chi^2	1.639	11.648
Pb	LogLikelihood	−22.286	−16.6
	p	0.645	0.003
	Chi^2	0.878	11.37
Zn	LogLikelihood	−128.179	−123.224
	p	0.195	0.007
	Chi^2	3.269	9.91

Supplementary Table 2. Result of Generalized Linear Model (GLZ) of parameters of soil.

Parameters		Area (Urban, Rural, Industrial)
Al	Wald Stat	2.955
	p	0.418
Ba	Wald Stat	7.012
	p	0.03
Ca	Wald Stat	2.148
	p	0.342
Cr	Wald Stat	8.083
	p	0.018
Cu	Wald Stat	2.775
	p	0.249
Fe	Wald Stat	7.015
	p	0.029
K	Wald Stat	8.409
	p	0.014
Mg	Wald Stat	10.441

	p	0.005
Mn	Wald Stat	5.438
	p	0.065
Na	Wald Stat	8.361
	p	0.015
Ni	Wald Stat	9.076
	p	0.01
Pb	Wald Stat	5.423
	p	0.066
Zn	Wald Stat	0.583
	p	0.747
humus, %	Wald Stat	0.505
	p	0.777
stability coefficient of humus	Wald Stat	0.148
	p	0.927
